# Engineering the Rhizosphere Microbiome with Plant Growth Promoting Bacteria for Modulation of the Plant Metabolome

**DOI:** 10.3390/plants13162309

**Published:** 2024-08-20

**Authors:** Maria J. Ferreira, Ana C. S. Veríssimo, Diana C. G. A. Pinto, Isabel N. Sierra-Garcia, Camille E. Granada, Javier Cremades, Helena Silva, Ângela Cunha

**Affiliations:** 1 CESAM and Biology Department, University of Aveiro, Campus de Santiago, 3810-193 Aveiro, Portugal; mjoaovf@ua.pt (M.J.F.); inatalia.sierra@ua.pt (I.N.S.-G.); hsilva@ua.pt (H.S.); 2 LAQV-REQUIMTE and Chemistry Department, University of Aveiro, Campus de Santiago, 3810-193 Aveiro, Portugal; carolinaana@ua.pt (A.C.S.V.); diana@ua.pt (D.C.G.A.P.); 3 Department of Genetics, Institute of Biosciences, Federal University of Rio Grande do Sul (UFRGS), Porto Alegre CEP 91501-970, RS, Brazil; camille.granada@ufrgs.br; 4 Interdisciplinary Center for Chemistry and Biology (CICA), University of A Coruña, 15071 A Coruña, Spain; javier.cremades@udc.es

**Keywords:** *Brevibacterium casei*, halophytes, bacterial consortia, plant-growth-promoting bacteria (PGPB), *Pseudomonas oryzihabitans*, plant metabolites, stress responses, UHPLC–MS, GC–MS, *Salicornia europaea*

## Abstract

Plant-growth-promoting bacteria (PGPB) have beneficial effects on plants. They can promote growth and enhance plant defense against abiotic stress and disease, and these effects are associated with changes in the plant metabolite profile. The research problem addressed in this study was the impact of inoculation with PGPB on the metabolite profile of *Salicornia europaea* L. across controlled and field conditions. *Salicornia europaea* seeds, inoculated with *Brevibacterium casei* EB3 and *Pseudomonas oryzihabitans* RL18, were grown in controlled laboratory experiments and in a natural field setting. The metabolite composition of the aboveground tissues was analyzed using GC–MS and UHPLC–MS. PGPB inoculation promoted a reconfiguration in plant metabolism in both environments. Under controlled laboratory conditions, inoculation contributed to increased biomass production and the reinforcement of immune responses by significantly increasing the levels of unsaturated fatty acids, sugars, citric acid, acetic acid, chlorogenic acids, and quercetin. In field conditions, the inoculated plants exhibited a distinct phytochemical profile, with increased glucose, fructose, and phenolic compounds, especially hydroxybenzoic acid, quercetin, and apigenin, alongside decreased unsaturated fatty acids, suggesting higher stress levels. The metabolic response shifted from growth enhancement to stress resistance in the latter context. As a common pattern to both laboratory and field conditions, biopriming induced metabolic reprogramming towards the expression of apigenin, quercetin, formononetin, caffeic acid, and caffeoylquinic acid, metabolites that enhance the plant’s tolerance to abiotic and biotic stress. This study unveils the intricate metabolic adaptations of *Salicornia europaea* under controlled and field conditions, highlighting PGPB’s potential to redesign the metabolite profile of the plant. Elevated-stress-related metabolites may fortify plant defense mechanisms, laying the groundwork for stress-resistant crop development through PGPB-based inoculants, especially in saline agriculture.

## 1. Introduction

Plants growing under natural field conditions often face a wide range of abiotic and biotic stresses that are not present in controlled settings. These stresses can significantly impact plant growth, physiology, and metabolic processes. The plant microbiome is currently recognized as a key player in the evolutionary and adaptive success of the plant host, acting through a complex network of stress responses and immune defenses [1]. In this context, halophytes and their microbiomes have been explored as model biological systems in order to unveil the connection between the intrinsic tolerance and the abiotic stress imposed by elevated salinity and the populations of plant-growth-promoting bacteria (PGPB) represented in the phytomicrobiome [2,3]. While having evolved specialized mechanisms to tolerate high salinity, such as compartmentalizing ions in the vacuoles, secreting salts through the glands, and maintaining water balance, halophytes are still subject to the combined effects of multiple abiotic and biotic stresses, limiting their growth and productivity [4,5]. Under these naturally challenging conditions, the selective recruitment of beneficial microbes is known to underlie the ecological success of these plants.

The rhizosphere, the zone of soil surrounding and influenced by the plant roots, is the main source of PGPB [6,7]. Therefore, engineering the rhizosphere microbiome with selected inoculants is regarded as an expedited tool to redesign the plant microbiome, with the final purpose of mitigating stress effects, activating defenses against infection, and enhancing productivity [6,7,8]. This approach is the conceptual ground for the development of microbe-based plant protection products, such as microbial biostimulants, biofertilizers, and biocontrol agents [9,10,11]. PGPB formulations are also becoming essential in enhancing the bioremediation potential of targeted plants, increasing their tolerance to heavy metals, and facilitating the solubility and mobility of soil contaminants [12]. Additionally, these formulations have been successfully used in soil desalination efforts [13].

The communication between the plant and the microbiome involves a complex network of biochemical signals and effectors. Inoculation with PGPB triggers metabolic reprogramming in plants, changing the levels of several primary and secondary metabolites [14,15]. PGPB inoculation can lead to an increased expression and accumulation of aromatic amino acids like phenylalanine and tryptophan, which serve as the precursors for the biosynthesis of defense-related compounds such as phenylpropanoids and indole alkaloids [16,17]. Additionally, PGPB can modulate the levels of TCA cycle intermediates, fatty acids, and other metabolites involved in plants’ primary metabolism, potentially redirecting resources towards producing specialized defense metabolites [17]. The activation of stress-protective pathways reshapes not only the plant metabolome, but also its elemental composition [18,19,20,21] and may ultimately add economic value to plants by widening their prospective uses and applications [22].

PGPB-induced metabolic changes have been observed across different plant species, including horticultural crops like tomato, lettuce, and onion, as well as ornamental plants like *Peperomia pellucida* [14,16,23,24]. The specific metabolic profiles induced by PGPB can vary depending on the bacterial strain, plant species, and environmental conditions, but generally result in enhanced plant growth, stress tolerance, and defense capabilities [18,25,26]. From a fundamental perspective, the patterns of enrichment in specific metabolites in response to microbiome engineering offer invaluable insights into the mechanisms of plant–bacteria interactions [27]. On a broader scale, understanding these PGPB-mediated metabolic alterations is crucial for developing sustainable agricultural and horticultural practices that leverage the natural abilities of beneficial microbes to improve plant performance under challenging field conditions.

The halophyte *Salicornia europaea* is known as an ecologically versatile and economically valuable plant and a promising candidate crop for saline soil agriculture and aquaponics systems. It also contains interesting and valuable secondary metabolites [8,28,29]. These unique characteristics have sparked considerable interest in the plant, leading to a growing number of studies exploring its biochemical potential, particularly of the aboveground tissues, for the pharmaceutical industry [30,31,32]. This interest in particular high-added-value compounds represented in the plant metabolome has highlighted the need for a deeper understanding of the microbiome–metabolome relations in this model halophyte.

In the last decade, important information has been gathered regarding the metabolite composition of *S. europaea*. Recent studies provide encouraging evidence that PGPB inoculation of *Salicornia europaea* boosts beneficial bacteria–plant interactions within the rhizosphere [33] and produces a significant impact on the plants’ primary metabolism, favoring biomass production [8]. However, these studies have primarily focused on microcosm conditions and on primary metabolism. In natural field environments, the combination of high salinity and sodicity creates a particularly stressful habitat that is challenging even for halophytes. These conditions compel plants to bolster their antioxidant and osmoprotective molecular mechanisms for survival [34]. The complex interplay between environmental factors and plant responses in the field may lead to different metabolic outcomes compared to controlled microcosm settings. Thus, we have hypothesized that *S. europaea* inoculation with PGPB might reshape the plant’s secondary biochemical profile. In this study, we aimed to investigate the impact of rhizosphere engineering with PGPB on the expression of stress resistance and growth-promoting secondary metabolites in *Salicornia europaea* L. under both controlled and field conditions.

## 2. Results

Gas-chromatography–mass-spectrometry (GC–MS) and ultra-high-performance liquid-chromatography–mass-spectrometry (UHPLC–MS) analyses of aboveground tissues have revealed differences in the metabolic profile between plants inoculated with halotolerant *Brevibacterium casei* EB3 and *Pseudomonas oryzihabitans* RL18, previously isolated from the roots (endosphere and rhizosphere) of *S. europaea* plants from the Portuguese coast [35], and non-inoculated plants, in both microcosm and field experiments. 

### 2.1. Plant Biochemical Profile—Microcosm Experiments

In the microcosm experiment, considering both GC– and UHPLC–MS analyses, a total of 62 compounds were identified, including 55 compounds in non-inoculated plants and 58 in inoculated plants (Supplementary Tables S1, S3 and S5). Due to a lack of a standard, seven compounds detected with GC–MS were not quantified (neophytadiene, oleanitrile, *N*,*N*-dimethylglycine, uridine, diacetone alcohol, glyceryl-glycoside, and 7-methylundec-4-ene). 

**Figure 1:**
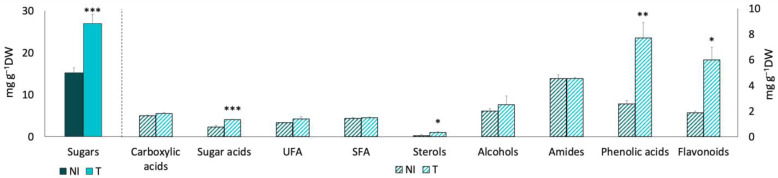
Phytochemical profile of non-inoculated and inoculated *Salicornia europaea* in controlled conditions (microcosm experiments), according to chemical families of compounds detected with GC–MS and UHPLC–MS. NI—non-inoculated plants; EB3 + RL18—plants inoculated with *Brevibacterium casei* EB3 and *Pseudomonas oryzihabitans* RL18. The columns represent the average of 3 biological replicates, and the error bars correspond to the standard error. The data obtained were compared by *t*-test (sugar acids, saturated fatty acids, alcohols, amides, and phenolic acids) or the Mann–Whitney test (unsaturated fatty acids, sterols, sugars, and flavonoids); significant differences are indicated by * (*p* < 0.05), ** (*p* = 0.004) and *** (*p* = 0.002) between the non-inoculated control and the test.

Sugars were the most prevalent compounds, while sterols were the least. The inoculated plants contained more sugars (32.6 ± 0.31 mg·g^−1^ DW), unsaturated fatty acids (UFA, 3.3 ± 0.86 mg·g^−1^ DW), and lower amounts of flavonoids (2.7 ± 0.05 mg·g^−1^ DW) compared to the non-inoculated control plants (15.5 ± 1.26 mg·g^−1^ DW, 1.6 ± 0.07 mg·g^−1^ DW, and 8.5 ± 0.45 mg·g^−1^ DW, respectively) (Figure 1).

A detailed characterization of the chemical composition (Supplementary Tables S1, S3 and S5) showed that the inoculated plants accumulated significantly higher concentrations of some carboxylic and sugar acids, namely acetic, citric, and tartaric acids, compared to the control plants. Conversely, the concentration of glyceric and ribonic acids was significantly lower in the test plants compared to the non-inoculated controls.

The fatty acid profile showed significant enrichment of the unsaturated oleic acid and the saturated stearic acid in the inoculated plants. Inoculation also resulted in an accumulation of sterol β-sitosterol, which was detected only in the inoculated plants. A wide variety of sugars was detected, among which the most represented were sucrose in the inoculated plants and D-glucose in the non-inoculated controls. Significant differences were observed only for xylose, which was absent in the inoculated plants, and sucrose, which was enriched in the latter.

The alcohols glycerol, myo-inositol, and butane-1,3-diol were enriched in the control plants (*p* < 0.05), whereas xylitol and 2-methylbutane-1,3-diol were detected exclusively in the inoculated plants.

The phenolic acid hydroxybenzoic acid and the flavonoids gallocatechin and catechin were found only in the non-inoculated plants (*p* < 0.05), which were also enriched in formononetin. In contrast, the phenolic acids caffeic acid and quinic acid and the flavonoid quercetin were detected only in the inoculated plants, which were also enriched in caffeoylquinic acids.

**Figure 2:**
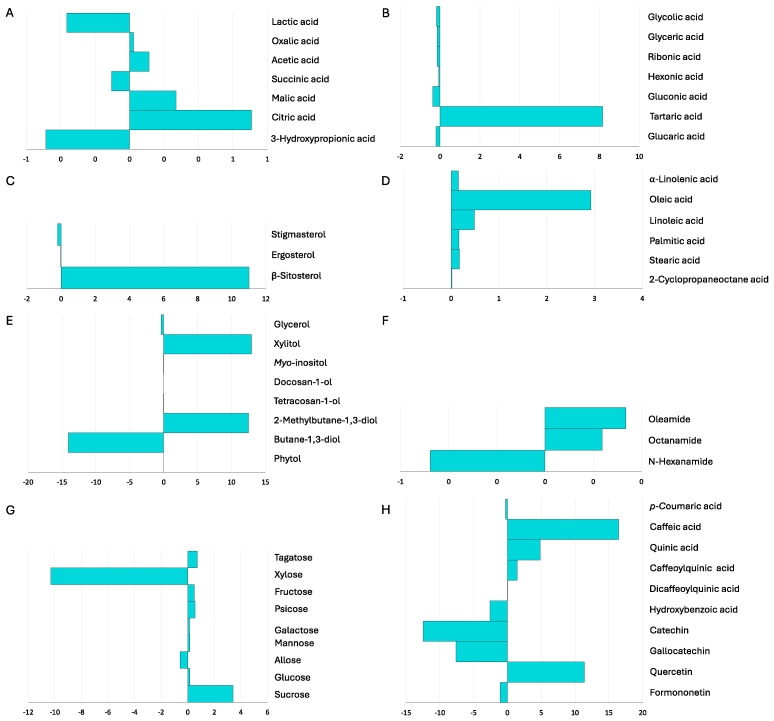
Response of *Salicornia europaea* secondary metabolism to inoculation with PGPB *Brevibacterium casei* EB3 and *Pseudomonas oryzihabitans* RL18 under controlled microcosm conditions. Fold changes are expressed as Log_2_ ((EB3 + RL18)/NI). (**A**)—Carboxylic acids; (**B**)—Sugar acids; (**C**)—Sterols; (**D**)—Fatty acids; (**E**)—Alcohols; (**F**)—Amides; (**G**)—Sugars; (**H**)—Phenolic compounds.

The fold changes in the concentration of metabolites for which significant differences between the inoculated and non-inoculated plants, expressed as Log_2_ ((EB3 + RL18)/NI), were detected are summarized in Figure 2. The heterogeneous response profiles to inoculation are visible within each group of metabolites, where positive and negative responses can be observed. The profile response of the sugar acids and flavonoids (especially catechin, gallocatechin, and formononetin) was negative, characterized by a decrease in these metabolites with inoculation, whereas the profile response of the carboxylic acids (malic, citric, and acetic acids), fatty acids (especially unsaturated fatty acids), sucrose, ß-sitosterol, phenolic acids, and quercetin was positive, representing an increase in these metabolites in the inoculated plants.

### 2.2. Plant Biochemical Profile—Field Experiments

In the field experiments, we identified 54 distinct compounds in the plant material using GC–MS and UHPLC–MS analysis, as detailed in Supplementary Tables S2, S4 and S6. Among these, 46 compounds were found in the non-inoculated plants, and 48 in the inoculated ones. However, four of the detected compounds—oleanitrile, glycine, glyceryl-glycoside, and 7-methylundec-4-ene—could not be quantified due to the unavailability of suitable standards.

**Figure 3:**
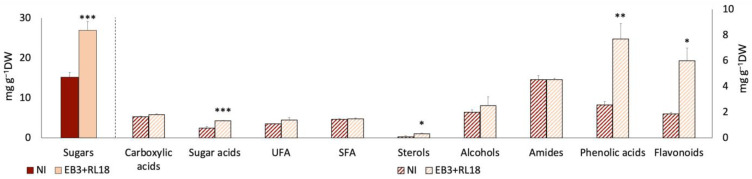
Phytochemical profile of non-inoculated and inoculated *Salicornia europaea* under field conditions, according to chemical families of compounds detected with GC–MS and UHPLC–MS. NI—non-inoculated plants; EB3 + RL18—plants inoculated with *Brevibacterium casei* EB3 and *Pseudomonas oryzihabitans* RL18. The columns represent the average of 3 biological replicates, and the error bars correspond to the standard error. The data obtained were compared by *t*-test (carboxylic acids, sugar acids, and amides) and the Mann–Whitney U test (saturated and unsaturated fatty acids, sterols, alcohols, and sugars); significant differences are indicated by * (*p* < 0.05), ** (*p* = 0.004) and *** (*p* = 0.002) between the control and the test.

In terms of the contribution to dry weight, sugars were the most important class of metabolites in the field-grown plants, as in the microcosm experiments, while sterols had the least contribution. The inoculated plants showed increased levels of sugars (26.9 ± 2.25 mg·g^−1^ DW), sugar acids (1.3 ± 0.02 mg·g^−1^ DW), sterols (0.3 ± 0.04 mg·g^−1^ DW), phenolic acids (7.7 ±1.19 mg·g^−1^ DW), and flavonoids (6.0 ± 0.98 mg·g^−1^ DW) when compared to their non-inoculated counterparts (15.2 ± 1.13, 0.8 ± 0.13, 0.1 ± 0.08, 2.6 ± 0.27, and 1.0 ± 0.12 mg·g^−1^ DW, respectively) (Figure 3).

The comprehensive analysis revealed that six carboxylic acids were consistently present in the inoculated and non-inoculated plants. Notably, ribonic acid, a sugar acid, was exclusive to the inoculated plants, whereas glyceric acid was detected only in the non-inoculated group. The fatty acid profile of the inoculated plants was significantly enriched with linoleic, palmitic, and lignoceric acids. The sterol analysis identified cholesterol and stigmasterol, with stigmasterol being significantly enriched in the inoculated plants (*p* < 0.05).

Higher concentrations of sugars, including fructose, psicose, mannose, allose, glucose, and sucrose, were detected in the inoculated plants, while ribose was unique to the controls. Sucrose remained the predominant sugar found across both conditions. Among the five alcohols detected, docosan-1-ol was more prevalent in the non-inoculated plants, with the other alcohols being present in similar concentrations, regardless of treatment. The amide pool showed that octanamide was exclusive to the inoculated plants, while dodecanamide was found only in the control plants. Unlike in the microcosm experiment, no terpenoids were detected in the field experiment. Glycine, although not quantified, was exclusively detected in the inoculated plants.

The phenolic acid analysis revealed that 3-*p*-coumaroylquinic acid and *p*-coumaric acid were only present in the non-inoculated plants, whereas hydroxybenzoic acid, quercetin, and apigenin were unique to the inoculated plants.

**Figure 4:**
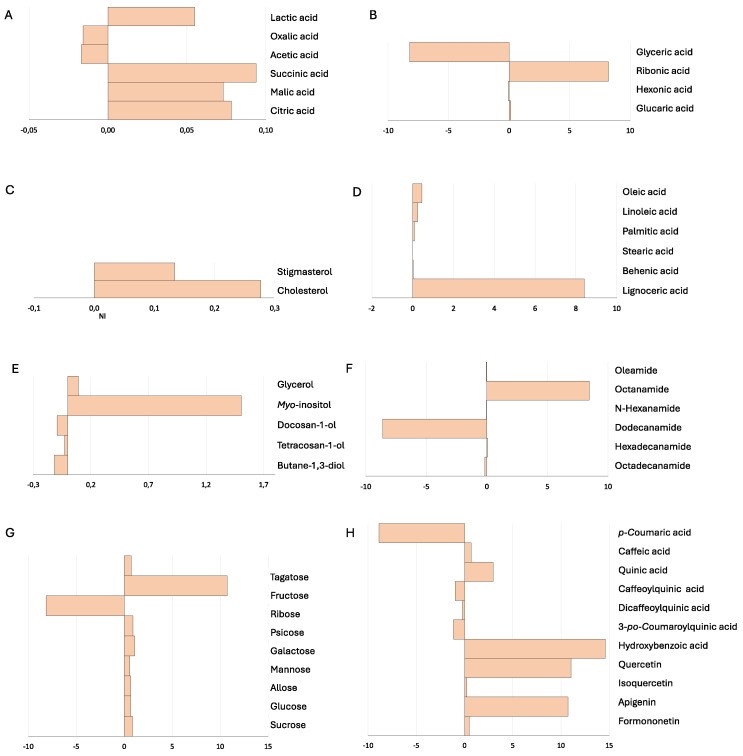
Response of *Salicornia europaea* secondary metabolism to inoculation with PGPB *Brevibacterium casei* EB3 and *Pseudomonas oryzihabitans* RL18 under field conditions. Fold changes are expressed as Log_2_ ((EB3 + RL18)/NI). (**A**)—Carboxylic acids; (**B**)—Sugar acids; (**C**)—Sterols; (**D**)—Fatty acids; (**E**)—Alcohols; (**F**)—Amides; (**G**)—Sugars; (**H**)—Phenolic compounds.

Figure 4 illustrates the fold changes in the concentration of metabolites, showing significant differences between the inoculated and the non-inoculated plants, expressed as Log_2_ ((EB3 + RL18)/NI). As in the microcosm experiments, inoculation led to increases and decreases in various metabolites. Flavonoids such as quercetin, apigenin, and formononetin, and phenolic acids like caffeic, quinic, and hydroxybenzoic acids, showed notable increases in the inoculated plants. Increases were also observed in fructose, myo-inositol, lignoceric acid, and oleic acid. Significant positive changes were recorded in carboxylic acids, including lactic, succinic, malic, and citric acids.

**Figure 5:**
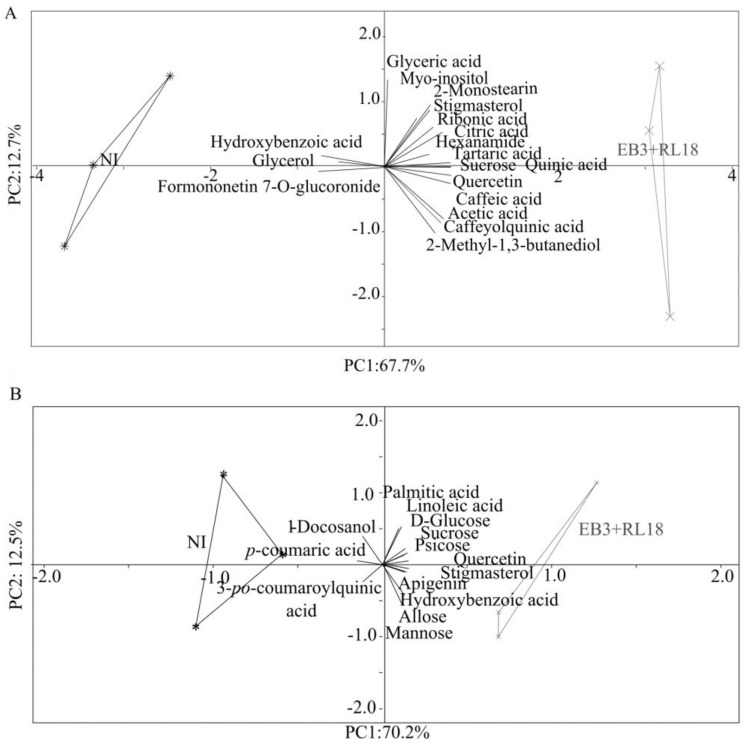
Principal component analysis (PCA) of (**A**) the microcosm experiment and (**B**) the field experiment. Changes in the metabolite composition of *Salicornia europaea* plants inoculated with *Brevibacterium casei* EB3 and *Pseudomonas oryzihabitans* RL18 (EB3 + RL18, gray stars) versus the corresponding non-inoculated controls (NI, black asterisks). Each symbol corresponds to an individual analyzed plant.

Principal component analysis (PCA) indicated that the two first axes explained 80.4% (Figure 5A) and 82.65% (Figure 5B) of the total data variability of the microcosm and the field experiments, respectively. In each experiment, the experimental conditions (the inoculated tests and the non-inoculated controls) clearly separate along axis one, which explains 67.7% (microcosm experiment) and 70.17% (field experiment) of the total variability, respectivley.

The metabolites sucrose, quercetin, tartaric acid, and caffeic acid were strongly correlated with the inoculated plants in the microcosm experiment, while quercetin, apigenin, psicose, and stigmasterol were more correlated with the inoculated field plants. In contrast, hydroxybenzoic acid and formononetin showed the highest correlation with the non-inoculated plants from the microcosm experiment.

## 3. Discussion

*Brevibacterium casei* EB3 and *Pseudomonas oryzihabitans* RL18, bacteria isolated from the roots (endosphere and rhizosphere) of *S. europaea*, have been identified as effective agents in enhancing the growth of *S. europaea*. Their influence is associated with a restructuring of the primary metabolic pathways, favoring biomass production [8]. Given the insights gained from the primary metabolism studies, significant alterations in secondary metabolism are anticipated following inoculation. The impact of bacterial inoculation on the profile of secondary metabolites in different ecological contexts provides valuable insights into plant responses, as well as the nutritional and pharmaceutical value of the inoculated plants. This has prompted the undertaking of the present study. The objective was to investigate the influence of growth conditions and co-inoculation with PGPB, specifically *B. casei* EB3 and *P. oryzihabitans* RL18, on the metabolite profiles of *Salicornia europaea*.

The phytochemical profile was characterized with GC–MS and UHPLC–MS analyses of extracts from the plant’s aerial part. As a general trend, in the microcosm experiments, the inoculated plants exhibited an enhanced sugar content and unsaturated fatty acids compared to the non-inoculated counterparts but had lower amounts of flavonoids. In the field conditions, in addition to containing more flavonoids, the inoculated plants were also enriched in phenolic acids, sugar acids, sterols, and sugars.

### 3.1. Metabolic Shifts and Plant Growth

PGPB are associated with several positive effects on plant health and phytochemical composition. In this study, both microcosm-grown and field plants inoculated with the selected PGPB exhibited enriched sugar content. Sugars, including sucrose, glucose, fructose, and trehalose, play diverse roles beyond energy provision, acting as signaling molecules that interact with phytohormones to enhance plant defense mechanisms and promote growth, sometimes prioritizing defense against stress over growth [36,37,38,39,40]. Elevated sucrose levels have been linked to delayed flowering, extending the vegetative phase and prolonging the harvest period for human consumption [41]. Additionally, sucrose exudation from plant roots serves as an osmoprotectant and regulates the beneficial rhizobacterial communities by alleviating water and salt stress [42,43,44].

Fructose, which also accumulated significantly in the inoculated field plants, enhances the plant’s ability to tolerate long-term stress, including chronic drought and high salinity, by activating the genes involved in defense mechanisms [45,46].

Elevated glucose levels, observed in the inoculated field plants, have been associated with an extended juvenile phase, with reduced growth rates [47]. This may help to explain the lack of growth enhancement published in a previous study [8] on the same plants, where no significant difference in growth was observed between the inoculated field plants and the control plants. *S. europaea* is an annual halophyte that accumulates the majority of its biomass during the vegetative phase, which lasts for approximately 9 weeks. Following this phase, the plant enters the reproductive stage, producing flowers and seeds. After seed dispersal, the plant dies [48]. Extending the juvenile phase results in less time for biomass production and overall growth.

Despite the fact that inoculation caused the field plants to accumulate psicose, allose, mannose, glucose, and sucrose, the microcosm-grown plants only showed significant sucrose accumulation. Nonetheless, a metabolic shift towards monosaccharide production was apparent in all inoculated plants. While sucrose showed a strong correlation with microcosm-grown inoculated plants, psicose exhibited a similar relationship with the inoculated field plants.

Studies on strawberries and rice have shown that inoculation led to increased plant growth and total phenolic content [20,21]. In particular, *Pseudomonas* sp. has revealed positive effects on plant growth and stress tolerance, altering the plant metabolite profile, both in greenhouse and field conditions [49,50,51,52,53,54].

In both environmental contexts, inoculation shifted the plant metabolism towards fatty acid production, influencing the total fatty acid content in the field-grown plants, particularly impacting the unsaturated fatty acids (UFAs) in microcosm conditions. UFAs play a crucial role as a carbon and energy source [55,56], while also protecting the photosynthetic machinery of plants that grow in saline conditions, thereby increasing stress resistance through membrane modification. Saturated fatty acids, in particular, play a crucial role in plant growth and seed formation [57].

Biomass production by inoculated microcosm-grown plants can also be associated with the accumulation of quercetin. This flavonoid is an effective antioxidant that strengthens the plant’s immune response [16]. It also enhances plant photosynthesis, alters soil chemistry, and attracts beneficial microbial populations; in addition, its growth promotion may be linked to the auxin transport following inoculation, resulting in the accumulation of this phytohormone in certain plant organs, impacting the plant growth and development processes [58,59,60].

Under controlled conditions, the inoculated plants exhibited higher concentrations of some organic acids, such as acetic acid, which can be utilized as an alternative carbon source by plant tissues [61]. Acetic and citric acids contribute to increased chlorophyll content and improved stress tolerance, ultimately promoting plant growth [62]. Moreover, inoculation with PGPB can affect the plant’s carboxylic acid profile and recruit beneficial bacteria for the root-associated microbiome [63]. Our analysis supports the hypothesis that, in both microcosm and field plants, a metabolic shift occurs, promoting the synthesis of these TCA cycle products, which underlies growth promotion.

### 3.2. Metabolic Shifts and Stress Tolerance

In addition to unpredictable and varying conditions such as weather, temperature, light, and water availability, the presence of competitors and pathogens [64] and soil/sediment characteristics in the growing field—marked by high values of electrical conductivity of both the sediment (129 dS m^−1^) and pore water (113 dS m^−1^), SAR (13.3), and ESP (17) [35]—sustain the scenario of harsher conditions in the field setting. Consequently, an accumulation of stress-related compounds, especially in the field-grown plants, was anticipated.

UFAs serve as precursors and intermediates in the biosynthesis of various bioactive compounds [65]. In the inoculated microcosm-grown plants, UFAs, particularly oleic acid, were significantly elevated. However, in the field-grown plants, the total UFA content did not differ from that found in the non-inoculated plants, although an enrichment in linoleic acid was noted. Despite this, the overall unsaturated fatty acid content and the ratio between the unsaturated and saturated fatty acids were reduced in the inoculated field plants, suggesting potential lipid peroxidation due to oxidative stress [66,67]. The stronger oxidative stress response by the inoculated plants is also linked to the absence of β-sitosterol and the presence of more saturated sterols, such as stigmasterol and cholesterol, suggesting increased β-oxidation under stressful field conditions [67]. Alpha-linolenic acid was not detected in the field-grown plants, contrary to the microcosm grown plants, which may signal stress-activated metabolic pathways. Jasmonic acid, derived from alpha-linolenic acid, plays a critical role in maintaining ionic balance, enhancing antioxidant defense, and modulating physiological processes to protect plants from stress-induced damage [68,69].

The inoculated field plants also show an enrichment in phenolic compounds. The accumulation of molecules such as hydroxybenzoic acid, quercetin, and apigenin, alongside a metabolic shift towards the production of formononetin, caffeic acid, and caffeoylquinic acid, not only suggests that the field plants were exposed to stressful conditions, but also supports the hypothesis that the inoculation induced a beneficial metabolic shift, providing the plants with a broader spectrum and increased amounts of protective compounds, consistent with the activation and diversification of stress protection mechanisms [70,71,72]. The increase in apigenin and quercetin levels in the inoculated field plants further reinforces the immune system, as they have outstanding antioxidant properties and act as ROS-quenching compounds [16], as other flavonoids do.

The increase observed in lignoceric acid in the inoculated field plants, a precursor of cell waxes and suberin, which act as a barrier of defense against environmental factors such as dehydration- and UV-induced stress [73], indicates that the inoculated plants are more effectively prepared to handle these stressors. This adaptation is likely crucial in a salt marsh environment, where dehydration plays an important role, especially with the frequent changes in hydration levels as the tide comes in and recedes.

The metabolite profile of the field plants additionally indicates a greater exposure to biotic stress, a conclusion further supported by our study’s exploration of phenolic acids. These compounds may elucidate the observed lignified appearance in the lower portion of the shoots of the field-grown plants compared to those grown in pots (Supplementary Figure S1). Phenolic acids, which are integral to processes such as plant–microbe symbiosis, allelopathy, and lignin cross-linking, may contribute to fortifying the cell walls against pathogen invasion [74]. The chemical structure of flavonoids allows for the formation of hydrogen bonds between these compounds and the nucleic acid bases of the pathogens, therefore, inhibiting gene expression and DNA/RNA synthesis [16].

The previous findings from Ferreira et al. (2023b) [75] regarding the presence of azelaic acid and the enrichment of their microbiome with pathogenesis-related orthologs in crop plants from the same location provide additional context for understanding the susceptibility of the field plants to biotic stress.

It has been proven that the plant’s metabolic response is clearly shaped by inoculation. The type and concentration of the specific metabolites produced after the induction of priming with PGPB are not only strain-specific, but also respond differently to different bacterial components, sometimes even by triggering the same metabolic pathways but with different outcomes in terms of the pool of secondary metabolites produced [14,16,23].

Several studies on PGPB–plant-host interactions have reported increased flavonoids, phenolic acids, fatty acids, and carboxylic acids following PGPB inoculation. Additionally, these studies indicate a redirection of the plant’s primary metabolism to support secondary metabolic pathways, thereby enhancing the production of specialized defense metabolites [16,17,24,76].

The inoculated microcosm-grown plants, despite the absence of visible stress symptoms, also accumulated some specific antioxidant compounds, such as caffeic acid, quinic acid, caffeoylquinic acids, and quercetin. This indicates that a similar, although more subtill, metabolic shift may have occurred in the microcosm conditions, equipping the plants with rapid responses to oxidative and or saline stress.

### 3.3. Biostimulation of Salicornia europaea with PGPB: Implications for Economic Value

Halophytes, such as *Salicornia europaea,* have been gaining recognition as valuable sources of bioactive compounds such as polyphenols and flavonoids, which are found in both their vegetative parts and oils [77,78,79]. These compounds not only bolster plant resilience, but also offer potential health benefits for humans. The stress-induced accumulation of phenolic compounds enhances the nutritional and nutraceutical value of these plants [80,81]. Inoculation resulted in an increase in rare sugars, such as psicose and allose, known for their anti-obesity activity [62]. The other compounds that were enriched in the inoculated plants, such as caffeoylquinic acids, are beneficial for human sugar metabolism [82]. Moreover, the inoculated plants contain more essential fatty acids like linoleic acid (ω-6) and alpha-linolenic acid (ω-3) and quercetin, which have various health benefits, including the regulation of cholesterol levels, and have antibacterial and anti-inflammatory effects [83,84,85,86,87,88].

The phenolic content and sterol content of the inoculated field-grown plants increased significantly compared to those of the non-inoculated controls. Sterols and phenolic compounds, such as caffeoylquinic acids, and the flavonoids formononetin, quercetin, and apigenin, enhance the anti-inflammatory and anticancer potential of the inoculated field plants [82,89,90,91]. Notably, the presence of specific metabolites like ergost-25-ene-3,5,6,12-tetrol and phenolic acids such as caffeoylquinic acids, caffeic acid, and formononetin may enhance the antimicrobial and neuroprotective properties of this plant [82,89,92].

## 4. Materials and Methods

### 4.1. Reagents

For GC–MS analysis, palmitic acid, oleic acid, linoleic acid, malic acid, succinic acid, β-sitosterol, cholesterol, eicosanol, glycerol, galactose, hexatriacontane, N,O-bis(trimethylsilyl)trifluoroacetamide (BSTFA), and trimethylsilyl chloride (TMSCl) were purchased from Sigma-Aldrich^®^ (Steinheim, Germany). Pyridine was purchased from Panreac Quimica SLU (Barcelona, Spain).

For UHPLC–MS analysis, ethanol and methanol were purchased from Sigma-Aldrich^®^ (Steinheim, Germany). Quercetin, (+)-catechin, isorhamnetin, luteolin, chlorogenic acid, kaempferol, and cinnamic acid were purchased from Extrasynthese (Lyon, France).

All reagents were p.a. grade.

### 4.2. Plant Inoculation and Growth

The methodologies used for seed bacterization, germination, and cultivation were previously described by [8]. In brief, the *Salicornia europaea* seeds collected in the production fields owned by a private company (Horta dos Peixinhos, Aveiro, Portugal) were co-inoculated with *Brevibacterium casei* EB3 and *Pseudomonas oryzihabitans* RL18 (EB3 + RL18), which display several plant-growth-promoting traits (Supplementary Table S7). These bacterial strains were isolated from the roots (rhizosphere and endosphere) of *Salicornia europaea* specimens from the Portuguese coast [35].

Individual strains were grown in Tryptic Soy Broth (TSB; Liofilchem, Roseto degli Abruzzi, Italy) supplemented with 25 g L^−1^ NaCl for 48 h in a rotary shaker (150 rpm, 30 °C ± 2 °C). After centrifugation, cells were collected and washed twice in a sterile saline solution (NaCl 9 g L^−1^). A third resuspension in sterile saline was adjusted to 10^8^ CFU mL^−1^ (OD_600_~1). The inoculum was prepared using an equal volume of each bacterial suspension (1:1 *v*/*v*). The seeds were surface disinfected (2 min immersion in 1 mL solution of 1:1 proportion of hydrogen peroxide (30%) and ethanol (96%), followed by rinsing three times with sterile distilled water), submersed in the inoculum solution for 2 h in a rotary shaker (150 rpm, 30 °C ± 2 °C), and then pelleted using low-speed centrifugation (5000× *g*; 2 min). The non-inoculated seeds (NI, control) were immersed in a sterile saline solution (NaCl 9 g L^−1^) under the same conditions.

Simultaneous trials were conducted using both inoculated and control seeds, encompassing a microcosm (under controlled conditions) and a field (under natural conditions) experiment. For the microcosm experiments, the seeds were dried in a laminar flow chamber and germinated in 1% agar plates in a Sanyo MLR 350 H Versatile Environmental Test Chamber (Moriguchi, Osaka, Japan) programmed for a 16/8 h light/dark regime for 15 days at 24 °C. The germinated plantlets were transplanted into plastic pots (5.5 cm high × 5.5 cm diameter), previously disinfected with 75% sodium hypochlorite solution, and rinsed several times in sterile distilled water. Each pot contained a similar weight of a 1:1 mixture of three-times-autoclaved salt marsh sediment (from the same field plot used for the field experiment) and perlite. Five pots from each condition (inoculated and control plants) were placed in shallow trays containing 20% Hoagland’s solution modified by the addition of 10 g L^−1^ marine salt. For two months, the plants were maintained in a removable outdoor plastic greenhouse, under natural temperature and sunlight, and, after that time, all pots were placed in a growth chamber (Fitoclima D1200, Aralab, Sintra, Portugal) under controlled conditions (16/8 h photoperiod, 20–25 °C temperature, 40% relative humidity, and 500 μmol m^−2^ s^−1^ photon flux density) for 35 days, until the end of the experiment (Supplementary Figure S2A,B).

In the field experiment, the seeds were sowed immediately after bacterization in the crop area (40°39′2″ N/8°38′42″ W), a former saltpan, which has been repurposed for *Salicornia europaea* crop cultivation since 2015 (Supplementary Figure S2C,D). The seeds were placed inside plastic rims (15 cm diameter) in rows of 10, separated by 50 cm. The sediment salinity was approximately 129 dS m^−1^ [35].

The reinoculation of the microcosm (pot) and field plants was performed by the application of 20 mL of either the bacterial solution mixture (inoculated plants) or sterile saline solution (control plants) every 15 days until the end of the experiment (5 months of growth).

### 4.3. Sample Preparation for Phytochemical Analyses

After five months of growth, three specimens (aboveground biomass) from each inoculation condition were collected for further analysis (Supplementary Figure S1). Each specimen was considered a biological replicate. The plant material was dried at 60 °C until a constant weight was achieved and ground to a fine powder using liquid nitrogen and a household electric grinder (Krups, Germany).

### 4.4. Silylation and Gas-Chromatography–Mass-Spectrometry (GC–MS) Analysis

Direct silylation of each of the 3 biological replicates was performed to increase the sensitivity of the GC–MS analysis. In a screw-cap glass tube, 10 mg of dried powder of each sample and replicate was mixed with 125 μL of pyridine, 125 μL of BSTFA, 25 μL of TMSCl, and 90 μL of the internal standard solution (hexatriacontane 1 mg mL^−1^). Dichloromethane was added up to a final volume of 1 mL. The mixture was incubated in a water bath at 70 °C for 40 min with continuous magnetic stirring. After cooling, the mixture was filtered through a 0.45 μm nylon filter (Membrane Solutions) and injected into a GC–MS QP2010 Ultra Shimadzu (Kyoto, Japan) with an A ZB—5 ms J & W (30 m × 0.25 mm × 0.25 μm) capillary column. The injector temperature was set at 320 °C, and the transfer line temperature was set at 200 °C. The samples were injected with a split ratio of 1:10, and helium was used as the carrier gas, with a flux of 1.19 mL min^−1^. The temperature of the column was maintained at 70 °C for 5 min and then increased by 4 °C per minute until 250 °C, followed by an increase of 2 °C per minute until 300 °C, which was maintained for 5 min, totaling 80 min. The mass spectrometer was operated in electronic impact (EI) mode with an energy of 70 eV, and data were collected at a rate of 1 scan s^−1^ over a range of *m*/*z* 50–1000. The identification of the components was carried out by direct comparison with the library entries of the plant spectra database (NIST14 Mass spectral and WILEY Registry™ of Mass Spectra Data). All reagents used were of high-grade quality, and the equipment was previously calibrated.

The internal standard method was applied, and the amount of each metabolite present in each replicate (3 biological replicates, each analyzed twice) was obtained from calibration curves acquired by the injection of known concentration solutions of each standard or with its TMS derivatives. The standard concentrations were chosen to guarantee the quantification of each compound in the samples by interpolating the calibration curve.

### 4.5. Extraction and Ultra-High-Performance Liquid-Chromatography–Mass-Spectrometry (UHPLC–MS) Analysis

Ethanol extracts of each of the 3 biological replicates were obtained by mixing 10 mg of dry-weight powder with ethanol p.a. (2 mL, two cycles of 24 h each) at room temperature. After the extracting solution was filtered twice with hydrophilic cotton, the solvent was evaporated using a HyperVAC VC2200 (Scappoose, USA) at 40 °C until the complete dryness of the mixture was obtained. Each dried ethanol fraction was weighed, and methanol p.a. was added to dilute the samples (final concentration 10 mg/mL). The solutions were filtered through a 0.2 μm nylon membrane (Whatman). Unlike the GC analysis, it was not necessary to add a standard solution, since the components are in a liquid state; moreover, gas dissipation does not occur in this analysis. Each biological replicate was then injected into the UHPLC–MS apparatus.

The UHPLC–MS analysis was performed using a Thermo Scientific Ultimate 3000RSLC (Dionex, Sunnyvale, CA, USA) equipped with a Dionex UltiMate 3000 RS diode array detector and coupled with a mass spectrometer. The column used was a Thermo Scientific Hypersil gold column (100 mm × 2.1 mm) with a particle size of 1.9 mm, and its temperature was maintained at 30 °C. The mobile phase comprised 0.1% formic acid (*v*/*v*), degassed and filtered before use. The flow rate was 0.2 mL/min. The solvent gradient started with 5% solvent over 14 min, followed by 40% solvent for 2 min, 100% over 7 min, and 5% over 10 min. The injection volume was 2 μL. The UV–vis spectral data were gathered in a range of 190–700 nm, and the chromatographic profiles were documented at 320 nm. The mass spectrometer was an LTQ XL linear ion trap 2D equipped with an orthogonal electrospray ion source (ESI). The equipment was operated in negative-ion mode with an electrospray ionization source of 5.00 kV and an ESI capillarity temperature of 275 °C. The full scan covered a mass range of 50–2000 *m*/*z*. Collision-induced dissociation MS/MS and MS^2^ experiments were simultaneously acquired for precursor ions. Each sample was analyzed separately under the same chromatography conditions.

The amount of each metabolite was calculated using the calibration curves acquired by the injection of a known concentration of solutions of each standard diluted in methanol p.a. The concentration of the standards was chosen to guarantee the quantification of each compound in the samples by interpolation.

### 4.6. Statistical Analyses

Normality was assessed using the Shapiro–Wilks test. The differences between the experimental conditions were evaluated using a *t*-test whenever normal distribution was verified. Non-normal distributions were evaluated using the Mann–Whitney test. Significant differences were always considered at *p* < 0.05.

All of the above-mentioned statistical analyses were performed using IBM SPSS statistics, version 28.0.1.1 (14) and Infostat version 2020e [93]. Principal component analysis was performed with Past 4.11 [94].

Log_2_ fold change in the two groups’ means was calculated using Microsoft^®^ Excel for Mac (version 16.83).

## 5. Conclusions

The inoculation of *Salicornia europaea* seeds with PGPB *Brevibacterium casei* EB3 and *Pseudomonas oryzihabitans* RL18 induced lasting effects in the plants’ metabolome. In the microcosm experiments, the accumulation of sugars, unsaturated fatty acids, citric and acetic acids, sterols, phenolic acids, and flavonoids due to the inoculation likely allowed the plants to produce biomass and overcome potential environmental stresses. The corresponding effects in the field-grown plants indicate that the attenuation of stress was the primary driver of the metabolic redesign induced by the inoculation with PGPB, which is supported by the accumulation of metabolites such as sucrose, unsaturated fatty acids, malic acid, phenolic acids, and flavonoids.

The results confirm that plant responses to biostimulation are strongly influenced by the ecological context and allow for a deeper understanding of the mechanisms and metabolites that explain the variability of the outcome of the engineering of the rhizosphere microbiome with PGPB. Customized approaches to biofertilization and biocontrol may, therefore, be necessary under different scenarios.

Some questions were raised regarding the distinct effects of PGPB inoculation in pot versus field plants. Specifically, future research should delve into understanding the specific biochemical pathways responsible for the distinct metabolite profiles observed in pot versus field plants after PGPB inoculation. Investigating the long-term effects of PGPB inoculation on plant health and productivity in both controlled and field conditions is crucial in order to determine the sustainability of such practices. Understanding how the interactions between PGPB and the native soil microbiome affect plant health and metabolite production is also vital. Furthermore, identifying the most effective methods for applying PGPB to maximize their benefits in different agricultural settings will be essential for practical applications. A combination of plant metabolomics and a comprehensive analysis of the plant microbiome may elucidate the mechanisms underlying stress attenuation and growth promotion. There is, however, encouraging evidence to suggest that the targeted accumulation of important bioactive compounds can be induced by PGPB-inoculation, which, in addition to increasing productivity, may contribute to the increased nutritional and nutraceutical value of the plant.

## Data Availability

The data that support the findings of this study are available from the corresponding author upon reasonable request.

## Supplementary Materials

The following supporting information can be downloaded at: https://www.mdpi.com/article/10.3390/plants13162309/s1, Figure S1. Specimens of *S. europaea*, representative of different inoculation conditions, at the end of the experiments. (**A**) Microcosm experiment; (**B**) Field experiment. Control – non-inoculated plants; EB3+RL18 – plants inoculated with *Brevibacterium casei* EB3 and *Pseudomonas oryzihabitans* RL18. Figure S2. Cultivation settings for *S. europaea*. (**A**) Microcosm experiment, outdoor greenhouse; (**B**) Microcosm experiment, Growth Chamber; (**C**) Field experiment (40°39′2″N/8°38′42″W), detail of the experimental plot (sowing); (**D**) Field experiment, 4 months after sowing. Table S1. Identified compounds on two *S. europaea* samples, non-inoculated plants (NI) and the inoculated plants with the bacterial inoculants *Brevibacterium casei* EB3 and *Pseudomonas oryzihabitans* RL18 (EB3+RL18), under controlled conditions. * Mean statistically significant differences at *p* < 0.05. Table S2. Identified compounds on *S. europaea* non-inoculated plants (NI) and inoculated plants with the bacterial inoculants EB3 *Brevibacterium casei* and RL18 *Pseudomonas oryzihabitans* (EB3+RL18), under field conditions. * Mean statistically significant differences at *p* < 0.05. Table S3. Characterization of phytochemical profiles of non-inoculated (NI) and inoculated *S. europaea* plants with the bacterial inoculants EB3 *Brevibacterium casei* and RL18 *Pseudomonas oryzihabitans* (EB3 + RL18), grown under controlled conditions by ultra-high performance chromatography-mass spectrometry (UHPLC-MS). Retention time (Rt; min.), wavelengths of maximum absorption in the visible region (λmax; nm), molecular ion ([M − H]^−^; *m*/*z*) and mass spectral data (MS^n^; *m*/*z*). Table S4. Characterization of phytochemical profiles of non-inoculated (NI) and inoculated *S. europaea* plants with the bacterial inoculants EB3 *Brevibacterium casei* and RL18 *Pseudomonas oryzihabitans* (EB3+RL18), grown under field conditions by ultra-high liquid chromatography-mass spectrometry (UHPLC-MS). Retention time (Rt; min.), wavelengths of maximum absorption in the visible region (λmax; nm), molecular ion ([M − H]^−^; *m*/*z*) and mass spectral data (MS^n^; *m*/*z*). Table S5. Phenolic composition (mg·g^−1^ DW) of non-inoculated (NI) and inoculated *S. europaea* plants with the bacterial inoculants EB3 *Brevibacterium casei* and RL18 *Pseudomonas oryzihabitans* (EB3 + RL18), grown under microcosm conditions, according to quantified compounds detected by UHPLC-MS. Table S6. Phenolic composition (mg·g^−1^ DW) of non-inoculated (NI) and inoculated *S. europaea* plants with the bacterial inoculants EB3 *Brevibacterium casei* and RL18 *Pseudomonas oryzihabitans* (EB3 + RL18), grown under field conditions, according to quantified compounds detected by UHPLC-MS. Table S7. Summary of the plant growth promotion activities and salt tolerance of the tested bacterial strains. References [95,96,97,98,99,100,101,102,103,104] are cited in the Supplementary Materials.
