# Supplementary material for: Engineering the Rhizosphere Microbiome with Plant Growth Promoting Bacteria for Modulation of the Plant Metabolome
Source: Plants (Basel). 2024 Aug 20;13(16):2309. doi: 10.3390/plants13162309 (PMC11360282; doi:10.3390/plants13162309)
Supplement: Supplementary file 1 [file plants-13-02309-s001.zip › plants-3163700-supplementary.pdf]

Engineering the Rhizosphere Microbiome with Plant Growth Promoting Bacteria for Modulation of the Plant Metabolome

Maria J. Ferreira [1], Ana C. S. Veríssimo [2], Diana C. G. A. Pinto [2], Isabel N. Sierra-Garcia [1], Camille E. Granada [3], Javier Cremades [4], Helena Silva [1] and Ângela Cunha [1,*]

[1] CESAM and Biology Department, University of Aveiro, Campus de Santiago, 3810-193 Aveiro, Portugal; mjoaovf@ua.pt (M.J.F.); inatalia.sierra@ua.pt (I.N.S.-G.); hsilva@ua.pt (H.S.)
[2] LAQV-REQUIMTE and Chemistry Department, University of Aveiro, Campus de Santiago, 3810-193 Aveiro, Portugal; carolinaana@ua.pt (A.C.S.V.); diana@ua.pt (D.C.G.A.P.)
[3] Department of Genetics, Institute of Biosciences, Federal University of Rio Grande do Sul (UFRGS), CEP 91501-970 Porto Alegre, RS, Brazil; camille.granada@ufrgs.br
[4] Interdisciplinary Center for Chemistry and Biology (CICA), University of A Coruña, 15071 A Coruña, Spain; javier.cremades@udc.es
* Correspondence: acunha@ua.pt

SUPPLEMENTARY FIGURES

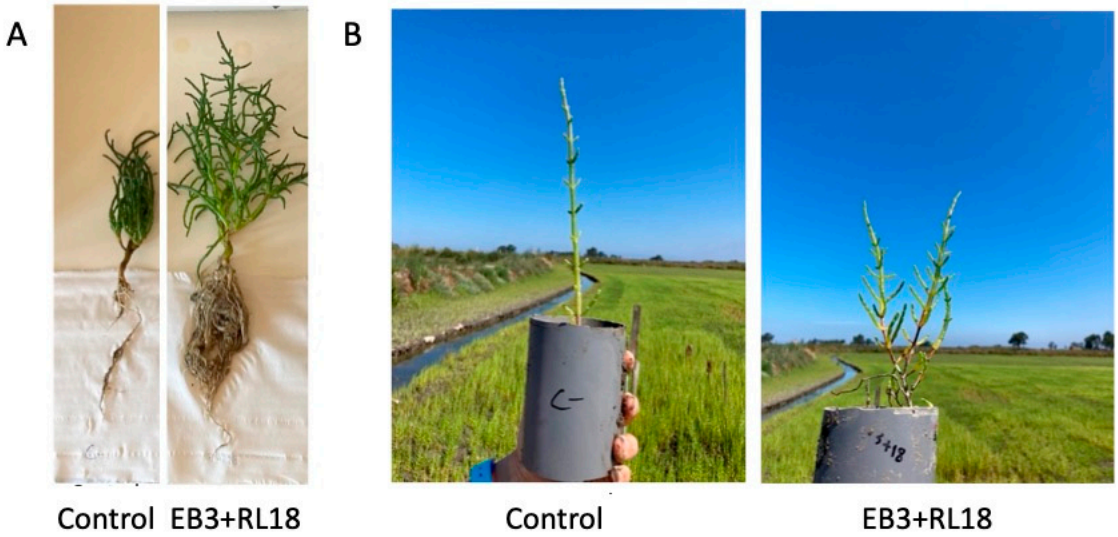

Supplementary Figure S1. Specimens of *S. europaea*, representative of different inoculation conditions, at the end of the experiments. A - Microcosm experiment; B - Field experiment. Control – non-inoculated plants; EB3+RL18 – plants inoculated with *Brevibacterium case*i EB3 and *Pseudomonas oryzihabitans* RL18.

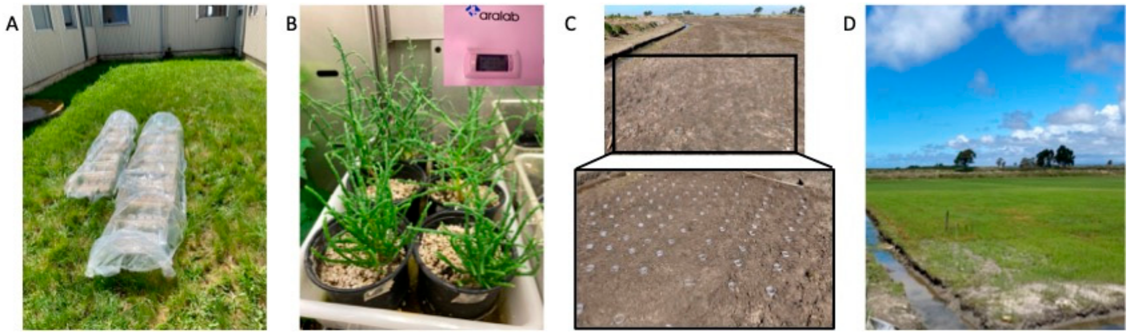

Supplementary Figure S2. Cultivation settings for *S. europaea*. A - Microcosm experiment, outdoor greenhouse; B - Microcosm experiment, Growth Chamber; C – Field experiment (40º39'2''N/8º38'42''W), detail of the experimental plot (sowing); D - Field experiment, 4 months after sowing

SUPPLEMENTARY TABLES

Supplementary Table S1. Identified compounds on two *S. europaea* samples, non-inoculated plants (NI) and the inoculated plants with the bacterial inoculants *Brevibacterium casei* EB3 and *Pseudomonas oryzihabitans* RL18 (EB3+RL18), under controlled conditions. * Mean statistically significant differences at $p < 0.05$

Rt	Identification [a]	NI**	EB3+RL18**
	Carboxylic acids		
8.98	Lactic acid	0.31±0.04	0.24±0.02
11.84	Oxalic acid	0.29±0.03	0,30±0.00
12.35	Acetic acid	0.22±0,00	0.24± 0.01 *
18.43	Succinic acid	0.29±0.02	0.27±0.01
24.18	Malic acid	0.39±0.05	0.46±0.01
33.54	Citric acid	0.23±0.01	0.37±0.01*
57.76	3-Hydroxypropionic acid	0.36±0.08	0.25±0.01
	Sugar acids		
9.62	Glycolic acid	0.22±0.01	0.19±0.01
19.00	Glyceric acid	0.23±0.01*	0.21±0.00
32.05	Ribonic acid	0.29±0.00*	0.26±0.00
34.52	Quininic acid	0.31±0.04	0.30±0.00
34.98	Hexonic acid	0.68±0.09	0.65±0.01
37.95	Gluconic acid	0.34±0.04	0.26±0.00
36.39	Tartaric acid	nd	0.29±0.01*
38.16	Glucaric acid	0.92±0,18	0.79±0.02
	Unsaturated fatty acids		

42.96	α-Linolenic acid	0.86±0.05	0.96±0.07
42.98	Oleic acid	0.30±0.00	2.26±0.95*
42.83	Linoleic acid	0.70±0.05	0.98±0.21
	Saturated fatty acids		
39.15	Palmitic acid	0.83±0.03	0.93±0.08
43.60	Stearic acid	0.33±0.02	0.37±0.02*
45.75	2-Cyclopropaneoctanoic acid	0.35±0.02	0.36±0.00
53.34	2-Monostearin	0.68±0.31	0.35±0.02
	Sterols		
66.00	Stigmasterol	0.19±0.04	0.17±0.01
67.509	(3β,5α,6β,12β)-Ergost-25-ene-3,5,6,12-tetrol	0.22±0.00	0.22±0.00
67.508	β-Sitosterol	nd	0.21±0.00*
	Sugars		
32.22	Tagatose	2.21±0.25	3.66±0.55
32.31	Xylose	1.27±0.00*	nd
34.24	Fructose	2.37±0.44	3.37±0.85
35.31	Psicose	2.32±0.42	3.42±0.53
35.61	Galactose	2.57±0.44	2.82±0.22
35.36	Mannose	2.07±0.10	2.30±0.17
35.62	Allose	1.99±0.40	1.34±0.01
37.65	Glucose	3.58±0.71	3.970±0.63
51.49	Sucrose	1.44±0.04	15.28±0.26*
	Alcohols		
17.08	Glycerol	1.30±0.13*	0.99±0.00
30.24	Xylitol	nd	0.80±0.00*
40.02	*Myo*-Inositol	0.84±0.01*	0.80±0.00
49.86	Docosan-1-ol	0.39±0.01	0.39±0.00
53.91	Tetracosan-1-ol	0.38±0.01	0.38±0.01
20.26	2-Methylbutane-1,3-diol	nd	0.57±0.14*
13.90	Butane-1,3-diol	1.71±0.00*	nd
35.08	Phytol	0.38±0.01	0.37±0.00
	Amides		
41.52	Oleamide	2.39±0.54	3.01±0.07
42.04	Octanamide	0.32±0.08	0.378±0.01
43.47	Hexanamide	0.67±0.00	0.481±0.09
	Terpenoids		
33.99	Neophytadiene	nq	nq
	Fatty nitriles		

39.87	Oleanitrile	nq	nq
	Amino acids		
6.36	*N*,*N*-Dimethylglycine	nq	nq
	Others		
47.58	Uridine	nq	nq
8.22	Diacetone alcohol	nq	nq
45.15	Glyceryl-glycoside	nq	nq
17.25	7-Methylundec-4-ene	nq	nq

RT = retention time; [a] All compounds possessing hydroxy groups are identified as the correspondent TMS derivatives. Compounds were identified by comparison with the GC-MS spectral libraries NIST14.lib and WILEY229.lib. nd=not detected; nq = not quantified. * significant differences (Mann-Whitney U test, $p<0.05$) between non-inoculated control and test plants. **Values in MV ± SE in mg of compound/g dry plant. MV = mean value; SE = standard error

Supplementary Table S2. Identified compounds on *S. europaea* non-inoculated plants (NI) and inoculated plants with the bacterial inoculants EB3 *Brevibacterium casei* and RL18 *Pseudomonas oryzihabitans* (EB3+RL18), under field conditions. * Mean statistically significant differences at $P < 0.05$

Rt	Identification [a]	NI**	EB3+RL18**
	Carboxylic acids		
8.98	Lactic acid	0.33±0.01	0.34±0.02
11.84	Oxalic acid	0.32±0.00	0.31±0.02
12.35	Acetic acid	0.32±0.00	0.32±0.01
18.43	Succinic acid	0.26±0.01	0.28±0.01
24.18	Malic acid	0.40±0.01	0.42±0.01
33.55	Citric acid	0.29±0.01	0.30±0.00
	Sugar acids		
19.00	Glyceric acid	0.30±0.00	nd
32.05	Ribonic acid	nd	0.30±0.01*
34.52	Quininic acid	0.56±0.00	0.28±0.01
34.98	Hexanoic acid	0.34±0.02	0.33±0.01
38.16	Glucaric acid	0.39±0.01	0.43±0.02
	Unsaturated fatty acids		
42.98	Oleic acid	0.52±0.02	0.72±0.18
42.83	Linoleic acid	0.57±0.02	0.68±0.07*
	Saturated fatty acids		
39.15	Palmitic acid	0.79±0.02	0.83±0.01*
43.60	Stearic acid	0.38±0.01	0.38±0.00
51.64	Behenic acid	0.32±0.01	0.33±0.01
55.94	Lignoceric acid	nd	0.34±0.01*
	Sterols		
66.00	Stigmasterol	0.25±0.00	0.28±0.01*
67.51	Cholesterol	0.24±0.00	0.29±0.00
	Sugars		
32.22	Tagatose	0.93±0.26	1.54±0.59
34.24	Fructose	nd	1.68±0.67
35.09	Ribose	0.29±0.05*	nd
35.31	Psicose	2.25±0.31	4.27±0.32*
35.61	Galactose	0.72±0.39	1.55±0.71
35.36	Mannose	1.45±0.02	2.16±0.15*
35.62	Allose	0.43±0.03	0.68±0.07*
37.65	Glucose	2.60±0.21	4.15±0.56*
51.49	Sucrose	8.20±0.79	14.92±1.80*

	Alcohols		
17.08	Glycerol	0.79±0.02	0.84±0.03
24.10	Butane-1,3-diol	0.53±0.02	0.49±0.00
40.02	*Myo*-Inositol	0.40± 0.02	1.13±0.73
49.86	Docosan-1-ol	0.38±0.00*	0.36±0.00
53.91	Tetracosan-1-ol	0.36±0.01	0.35±0.00
	Amides		
41.52	Oleamide	4.16±0.30	3.95±0.06
42.04	Octanamide	nd	0.35±0.00
42.06	Dodecanamide	0.38±0.00	nd
42.07	Hexadecanamide	0.37±0.02	0.38±0.00
43.47	Hexanamide	0.34±0.02	0.33±0.01
45.80	Octadecanamide	0.42±0.00	0.38±0.01
	Fatty nitriles		
39.87	Oleanitrile	nq	nq
	Amino acids		
6.36	Glycine	nd	nq
	Others		
45.15	Glyceryl-glycoside	nq	nq
17.25	7-Methylundec-4-ene	nq	nq

RT = retention time. [a] All compounds possessing hydroxy groups are identified as the correspondent TMS derivatives. Compounds were identified by comparison with the GC-MS spectral libraries NIST14.lib and WILEY229.lib. nd= not detected; nq = not quantified. * significant differences (Mann-Whitney U test, $p<0.05$) between non-inoculated control and test plants. **Values in MV ± SE in mg of compound/g dry plant. MV = mean value; SE = standard error

Supplementary Table S3. Characterization of phytochemical profiles of non-inoculated (NI) and inoculated *S. europaea* plants with the bacterial inoculants EB3 *Brevibacterium casei* and RL18 *Pseudomonas oryzihabitans* (EB3+RL18), grown under controlled conditions by ultra-high performance chromatography-mass spectrometry (UHPLC-MS). Retention time (Rt; min.), wavelengths of maximum absorption in the visible region (λmax; nm), molecular ion ($[M-H]^-$; m/z) and mass spectral data (MS^n; m/z).

Rt	λ max (nm)	$[M-H]^-$	MS^2	Assigned Identification	NI[a]	EB3+RL18[a]	Reference
1.41	203; 272; 368;484	377	341(100); 215(11)	Caffeic acid hexoside	nd	0.507± 0.011	[95]
1.42	203;309; 357; 368	161	93 (33); 161 (100); 134(18); 117(21); 133(7); 69(4)	*p*-Coumaric acid	0.50±0.01	nd	[95]
1.53	255; 281; 319; 375	161	93 (30) 161(100) 133(13) 117(14) 134(6)	*p*-Coumaric acid	0.38±0.02	0.71±0.29	[95]
1.75	205; 271; 309; 356	289	145; 159; 160; 173	Catechin[b]	2.80±0.04	nd	[96]

1.76	204; 271; 309; 368; 421	191	111 (100); 147 (45) 173 (35) 191 (5)	Quinic acid	nd	0.28±0.01	[97]
1.90	201; 208; 213; 220; 225; 238; 261; 280	289	243; 245; 203; 205	(epi)Catechin [b]	2.74±0.41	nd	[96]
1.91	203; 208; 244; 282	295	251(100); 133(15); 227(8); 179 (4)	Caffeic acid derivative	nd	0.39±0.06	[98]
5.15	198; 211	353	191 (100); 179(51); 173(4); 135(6)	5-Caffeoylquinic acid	nd	0.02±0.01	[98]
7.99	309; 331; 343	353	191 (100); 179(5); 173 (0,45); 135 (0.56)	Caffeoylquinic acid	0.06±0.00	0.10±0.02	[98]
8.44	310; 336	137	137	Hydroxybenzoic acid	0.03± 0.00	nd	[99]

8.44	285; 301; 324; 342	353	135(5); 173(100); 179(53); 191(17)	Caffeoylquinic acid	nd	0.04±0.01	[98]
10.64	310; 336; 356	443	267 (100); 411 (16); 425 (16); 249 (12); 193 (8); 175 (0,5); 161(1)	Formononetin7-*O*-glucoronide	0.01±0.00	0.02±0.00	[97]
10.88	314	137	137	Hydroxybenzoic acid	0.03±0.00	nd	[99]
10.89	296; 309; 324	163 (80); 179(40)	[163]:163 (100); 136 (43); 135 (14); 122 (5); 121 (29); 119 (70); 109 (1); 95 (3) [179]: 179; 161; 135	Caffeic and *p*-coumaric acids	nd	nq	
11.46	190	305	278 (100); 287(95); 262 (97); 259(81); 178(32)	Gallocatechin[b]	1.46±0.01	nd	[72]

11.62	190	609,5 (100) 305	[305]: 261(100); 243; 237;287; 278	Gallocatechin [b]	1.45± 0.01	nd	[100]
11.85	317; 340; 358	497 (100)	---	Unknown	nq	nd	
12.00	259; 314; 335	353	191; 263; 273; 291; 307; 309; 335; 353	Caffeyolquinic acid	0.06±0.01	nd	[98]
12.02	261; 296; 318; 345; 356	463	301(100); 300(14); 343(2);	Quercetin hexoside	nd	1.32±0.02	[101]
12.55	259; 267; 357	549 (100); 505(34); 353 ;179	[353]:191(100) 179(43) 173 (12) 335 (5)	Dicaffeoylquinic acid	0.04±0.01	nd	[102]
12.56	256; 315; 337	549(100) 505 (46)	[549]: 385(100); 383(69) [505]: 301 (100); 300 (36	Quercetin derivative	nd	1.35±0.02	[101]

12.74	312; 331; 343; 351; 358	515	353 (100); 335(11);299; 191(4); 179(5);173 (9)	Dicaffeoylquinic acid (1,4)	0.05±0.01	0.07±0.01	[102]
12.97	327; 340; 350; 385	515	353 (100); 335 (1); 191 (1); 179 (1); 173 (<1)	Dicaffeoylquinic acid (1,3)	0.05±0.01	0.09±0.02	[102]
13.62	354; 524; 547	515	353(100); 335 (4); 179 (2); 191 (0.75); 173 (4.4)	Dicaffeoylquinic acid	0.06±0.01	0.09±0.02	[102]
16.08	314; 334; 384	619	443 (100); 267 (3); 381(2); 425 (2); 193(1); 211 (1); 242(1)	Formononetin 7-*O*-glucoronide	0.01±0.00	0.01±0.00	[97]

[a] mg of compound/g plant dry weight, using three replicates for the error calculation; nq - not quantified due to the presence of an unknown compound or due to co-elution of two compounds. [b] standard solution documented at 280 nm; nd – not detected

Supplementary Table S4. Characterization of phytochemical profiles of non-inoculated (NI) and inoculated *S. europaea* plants with the bacterial inoculants EB3 *Brevibacterium casei* and RL18 *Pseudomonas oryzihabitans* (EB3+RL18), grown under field conditions by ultra-high liquid chromatography-mass spectrometry (UHPLC-MS). Retention time (Rt; min.), wavelengths of maximum absorption in the visible region (λmax; nm), molecular ion ($[M\text{-}H]^-$; m/z) and mass spectral data (MS^n; m/z).

Rt	λ max (nm)	[M-H]	MS^2	Assigned Identification	NI[a]	EB3+RL18[a]	Reference
1.42	205; 273	377(100) 215	[377]: 341(100); 215(12); 179(<1) [215]: 179 (100)	Caffeic acid derivative	0.48±0.04	0.54±0.06	[95]
1.53	255(100); 281 (80); 319 (40)	377	341(100); 215(10); 179(<1)	Caffeic acid derivative	0.76±0.35	1.44±0.56	[95]
1.63	203;309	163 (100)	163(100)	p-Coumaric acid	0.47±0.05	nd	[95]
1.76	205	191(100)	111 (100); 147 (22);173 (32)	Quinic acid	0.19±0.09	2.64±0.48	[97]
1.90	202; 208 214	137	137(100); 93	Hydroxybenzoic acid	nd	2.47±0.28	[99]

5.16	195; 204; 220; 226	353	191(100); 179(43); 173(3); 135(6)	3-Caffeyolquinic acid	0.03±0.01	0.02±0.01	[102]
7.98	243; 313; 326; 343	353	179 (5); 191(100)	3-Caffeyolquinic acid	nd	0.19±0.1	[102]
8.06	243; 313; 326; 343	353	179 (5); 191(100)	5-Caffeoylquinic acid	0.26±0.04	nd	[102]
8.44	276; 303; 327; 338	353	173 (100); 179 (48); 191(25); 135 (1.6)	4- Caffeoylquinic acid	nd	0.05±0.03	[102]
10.66	311; 333	443	193 (9); 249 (11); 253(3); 267(100); 283 (4);305(2); 381(3)	Formononetin 7-*O*-glucoronide	0.01±0.01	nd	[72]
10.89	282; 310	191	127; 147; 149; 163; 164; 173; 191	Quinic acid	nd	0.04±0.01	[97]
10.91	283; 312; 345;	163 (100); 455 (70) 337 (55)	[163]163(100); 136(99 121 (64)	3-*p*-Coumaroylquinic acid	0.02±0.01	nd	[98]

			[337]: 191 (100); 163; 175; 134 [455]: 275(100) 323; 313; 261				
11.87	256; 268 318	497	[497]: 451(100); 351; 415 [451]: 225 (100); 433; 367	Unknown	nd	nq	
12.02	256; 320; 352	463	301 (100) 300(20)	Isoquercetin	1.86±0.12	2.19±0.43	[103]
12.57	256	549 (100); 505 (47)	[549]: 270; 369; 421; 505 (100); 511; 531; 540 [505]: 301 (100); 300 (31); 463 (31)	Quercetin derivative	nd	2.09±0.40	[103]
12.87	242; 270; 304; 330	515(100); 353(40)	353 (100)	Dicaffeyolquinic acid	0.26±0.04	0.18±0.08	[102]

13.52	250; 320; 333	515 (100)	353 (100); 203 (12); 191 (0,66;) 299 (13); 317 8); 335 (100); 179(3); 173 (3)	Dicaffeyolquinic acid	0.09±0.03	0.12±0.05	[104] [102]
13.90	256; 294; 307; 324; 338; 351	563	250(73); 316(100); 383 (54); 431; 477 (73)	Apigenin derivative	nd	1.69±0.16	[99]
15.47	253; 294; 300; 318; 331; 343	179.17	[179]:135(100); 152; 170	Caffeic acid	nd	0.01±0.00	[95]
16.12	253; 282; 294; 300	619	443(100); 267 (10); 425(4); 505 (4)	Formononetin 7-*O*-glucoronide	nd	0.02±0.00	[97]

[a] mg of compound/g plant dry weight, using three replicates for the error calculation; nq - not quantified due to the presence of an unknown compound; nd – not detected

Supplementary Table S5. Phenolic composition (mg·g^{-1} DW) of non-inoculated (NI) and inoculated *S. europaea* plants with the bacterial inoculants EB3 *Brevibacterium casei* and RL18 *Pseudomonas oryzihabitans* (EB3+RL18), grown under microcosm conditions, according to quantified compounds detected by UHPLC-MS.

	Compound	Inoculation Condition	
		NI	EB3+RL18
Phenolic acids	*p*-coumaric acid	0.9±0.04	0.7±0.29
	Caffeic acid	0.0±0.00	0.9±0.05*
	Quinic acid	0.0±0.00	0.3±0.01*
	Caffeoylquinic acid	0.1±0.01	0.2±0.03*
	Dicaffeoylquinic acid	0.3±0.03	0.2±0.04
	Hydroxybenzoic acid	0.1±0.00*	0.0±0.00
Flavonoids	Catechin	5.5±0.44*	0.0±0.00
	Gallocatechin	2.9±0.02*	0.01±0.00
	Quercetin	0.0±0.00	2.7±0.04*
	Formononetin	0.02±0.00	0,01±0,00

NI - non-inoculated plants (n=3); EB3+RL18 – plants inoculated with *Brevibacterium casei* EB3 and *Pseudomonas oryzihabitans* RL18 (n=3). Values represented the mean ± standard error. * indicates significant statistical differences between treatments (t-test, $p<0.05$).

Supplementary Table S6. Phenolic composition (mg·g^{-1} DW) of non-inoculated (NI) and inoculated *S. europaea* plants with the bacterial inoculants EB3 *Brevibacterium casei* and RL18 *Pseudomonas oryzihabitans* (EB3+RL18), grown under field conditions, according to quantified compounds detected by UHPLC-MS.

	Compound	Inoculation Condition	
		NI	EB3+RL18
Phenolic acids	*p*-coumaric acid	0.5±0.05*	0.0±0.00
	Caffeic acid	1.2±0.38	2.0±0.55
	Quinic acid	0.2±0.09	1.5±0.76
	Caffeoylquinic acid	0.2±0.10	0.1±0.09
	Dicaffeoylquinic acid	0.3±0.03	0.3±0.12
	3-*po*-Coumaroylquinic acid	0.02±0.01*	0.0±0.00
	Hydroxybenzoic acid	0.0±0.00	2.5±0.28*
Flavonoids	Quercetin	0.0±0.00	2.1±0.40*
	Isoquercetin	1.9±0.12	2.2±0.43
	Apigenin	0.0±0.00	1.7±0.16*
	Formononetin	0.01±0.01	0.02±0.004

NI - non-inoculated plants (n=3); EB3+RL18 – plants inoculated with *Brevibacterium casei* EB3 and *Pseudomonas oryzihabitans* RL18 (n=3). Values represented the mean ± standard error. * indicates significant statistical differences between treatments (t-test, $p<0.05$).

Supplementary Table S7. Summary of the plant growth promotion activities and salt tolerance of the tested bacterial strains [35]

Isolates				Limit Salt Tolerance (g L^{-1})	Extracellular Enzymes					Plant-Growth Promoting Traits					
Code	Site	Source	Identification		Q	A	L	P	C	P-solubilization	Siderophore	ACC deaminase ($nm.mg^{-1}.h^{-1}$)	IAA (μgmL^{-1})	N-fixation	EPS (OD_{540})
EB3	Av3	E	*Brevibacterium casei*	100	−	−	−	+	+	−	+	+ (17.63 ± 1.35)	10.87 ± 0.48	+	1.24 ± 0.03
RL18	Tg	R	*Pseudomonas oryzihabitans*	100	−	+	−	−	+	+	+	+* (44.09 ± 4.33)	39.55 ± 1.01	−	0.40 ± 0.01

E—Endosphere; R—Rhizosphere; Q—Chitinase; A—Amylase; L—Lipase; P—Protease; C—Cellulase; Av3 - Boco (Aveiro); Tg – Tagus Estuary (Lisbon); + positive; − negative; +* visible growth on solid DF +ACC medium.
